# Catching moving targets: cancer stem cell hierarchies, therapy-resistance & considerations for clinical intervention

**DOI:** 10.1186/s12943-017-0601-3

**Published:** 2017-02-23

**Authors:** Claudia Gasch, Brendan Ffrench, John J. O’Leary, Michael F. Gallagher

**Affiliations:** 1 Department of Histopathology, University of Dublin, Trinity College, Central Pathology Laboratory, St James’s Hospital, Dublin 8, Dublin, Ireland; 2 Coombe Women and Infant’s Hospital, Dublin 8, Dublin, Ireland

## Abstract

It is widely believed that targeting the tumour-initiating cancer stem cell (CSC) component of malignancy has great therapeutic potential, particularly in therapy-resistant disease. However, despite concerted efforts, CSC-targeting strategies have not been efficiently translated to the clinic. This is partly due to our incomplete understanding of the mechanisms underlying CSC therapy-resistance. In particular, the relationship between therapy-resistance and the organisation of CSCs as Stem-Progenitor-Differentiated cell hierarchies has not been widely studied. In this review we argue that modern clinical strategies should appreciate that the CSC hierarchy is a dynamic target that contains sensitive and resistant components and expresses a collection of therapy-resisting mechanisms. We propose that the CSC hierarchy at primary presentation changes in response to clinical intervention, resulting in a recurrent malignancy that should be targeted differently. As such, addressing the hierarchical organisation of CSCs into our bench-side theory should expedite translation of CSC-targeting to bed-side practice. In conclusion, we discuss strategies through which we can catch these moving clinical targets to specifically compromise therapy-resistant disease.

## Background

Tumours are heterogeneous collections of cells, only some of which are capable of initiating tumourigenesis. In many different types of malignancy, these ‘tumour-initiating’ cells have been shown to display the stem cell-like properties of self-renewal, differentiation and the development of (malignant) tissues. This has led to tumour-initiating cells being collectively referred to as ‘Cancer Stem Cells’ (CSCs), and interest in targeting cancer stemness as a clinical strategy. CSCs have been shown to be highly-resistant to conventional cancer therapies such as chemotherapy and radiotherapy. While the targeting of CSC mechanisms has been shown to reduce therapy-resistance in many cell culture models, this has not been successfully translated to the clinic. In this review we will discuss successes and limitations in targeting CSC therapy-resistance mechanisms. We will argue that clinical-failure in this area may be partly due to a poor understanding of the plastic nature of the complex hierarchies into which CSCs are organised in vivo*.* Finally, we will conclude by arguing that clinical translation will be hastened by an appreciation of therapy-resistant CSC populations as moving, rather than fixed clinical targets.

## Stem cells, hierarchies, development, growth and repair

Stem cells (SCs) are defined as cells that can self-renew, produce different cell types during a cell division process known as ‘differentiation’, and re-generate the tissues from which they were generated [Reviewed in 1]. These properties are not shared by non-SCs [2]. SCs have the capacity for long-term proliferation in the undifferentiated state to perpetuate the SC pool throughout life (self-renewal). Depending on the body’s requirements, SCs can produce two undifferentiated cells through symmetrical self-renewal or two differentiated cells through symmetrical differentiation. Additionally, SCs often produce one undifferentiated cell and one differentiated cell simultaneously, in a process referred to as ‘asymmetric division’. The function of asymmetric division is to retain the pool of self-renewing cells while producing differentiating cells [3–5]. SCs use extensive rounds of self-renewal and differentiation to produce *de novo* tissues in the embryo and for growth and repair of tissues post-embryonically.

SCs are primarily characterised by their potency, a term used to refer to the number of cell and tissue types they can produce through differentiation. SCs are broadly categorised as Embryonic SCs (ESCs) and adult SCs. ESCs are found in the inner cell mass of the developing blastocyst and their primary function is to produce the tissues that compromise the body [6–8]. This property is referred to as pluripotency, which is defined as the ability to produce cells representative of all three germ layers (endoderm, mesoderm and ectoderm [9]). In contrast, adult SCs are located within specific niches in each adult tissue and function to produce new cells for growth and repair. Adult SCs are generally multipotent, which refers to their ability to generate several related cell types of relevance to their location. The best studied examples of the adult SC are the bone marrow SCs (BMSCs) of which there are two types: haematopoietic SCs, which produce the different types of blood cell, and mesenchymal stem/stromal cells (MSCs), which produce bone-related structural cells such as adipocytes, chondrocytes and osteoblasts [10].

In recent years it has become clear that SCs produce their differentiated progeny through one or more intermediaries known as (‘committed’) ‘Progenitors’. Progenitors are themselves SCs (can self-renew and differentiate), and are the work horses of tissuegenesis. However, progenitors are less potent than the parent SC that produces them, and in healthy tissues have a more limited proliferation potential [Reviewed in 1]. The concept of hierarchical arrangement of SCs was first described in bone marrow research. It is now known that HSCs and MSCs generate their repertoire of cell types through a number of intermediaries [11–14]. For example HSCs produce lymphoid and myeloid progenitors, which respectively develop to produce lymphocytes and myeloid cells such as red blood cells, neutrophils and macrophages. The Stem-Progenitor-Differentiated cell model has complicated SC analysis and, in particular, the identification and isolation of novel SCs. This is because it is now understood that most tissues contain multiple different SC types acting independently and inter-dependently. Unfortunately for CSC research, tumour tissue is similarly complicated, which has hindered clinical translation of CSC-targeting.

## Cancer stem cells, hierarchies and tumourigenesis

The concept of the CSC dates back to the study of the gonadal tumour ‘Embryonal Carcinoma’ (EC) by pathologists in the late 1890s [15, 16]. In describing the tissues within ECs as a disorganised caricature of the embryo, it was proposed that these tumours developed from ESC-like pluripotent cells. With the development of immune-compromised animal models in the 1960s, Kleinsmith and Pierce [9] showed that a single EC (stem) cell, the malignant counterpart of an ESC [15, 16], was sufficient for tumourigenesis. Although the term CSC was not used at the time, this was the first experimental proof of a CSC, and was pivotal to the Leukaemia SC (LSC) work that led to the declaration of the CSC theory and the current intense interest in CSCs. EC research was complimented by extensive LSC research, where the term CSC was first described. Following identification of LSCs by Bonnet and Dick [17] as the tumour-initiating cells of acute myeloid leukaemia, CSCs were later isolated from solid tumours, such as breast cancer and brain tumours [18, 19]. Since these seminal publications, CSCs have been isolated from many solid cancers, including lung, colon, prostate, ovarian cancer and melanoma, among others [20–24]. It is now well-established that tumour-initiating cells from many, if not all, malignancies share some of the properties of SCs [1, 25, 26]. Today, CSCs are defined by the SC properties of self-renewal, differentiation and the ability to efficiently (from low cell numbers) re-generate their original malignancy in vivo. Additionally, CSCs are known to resist standard interventions such as chemotherapy and radiotherapy [Reviewed in 27].

**Fig. 1:**
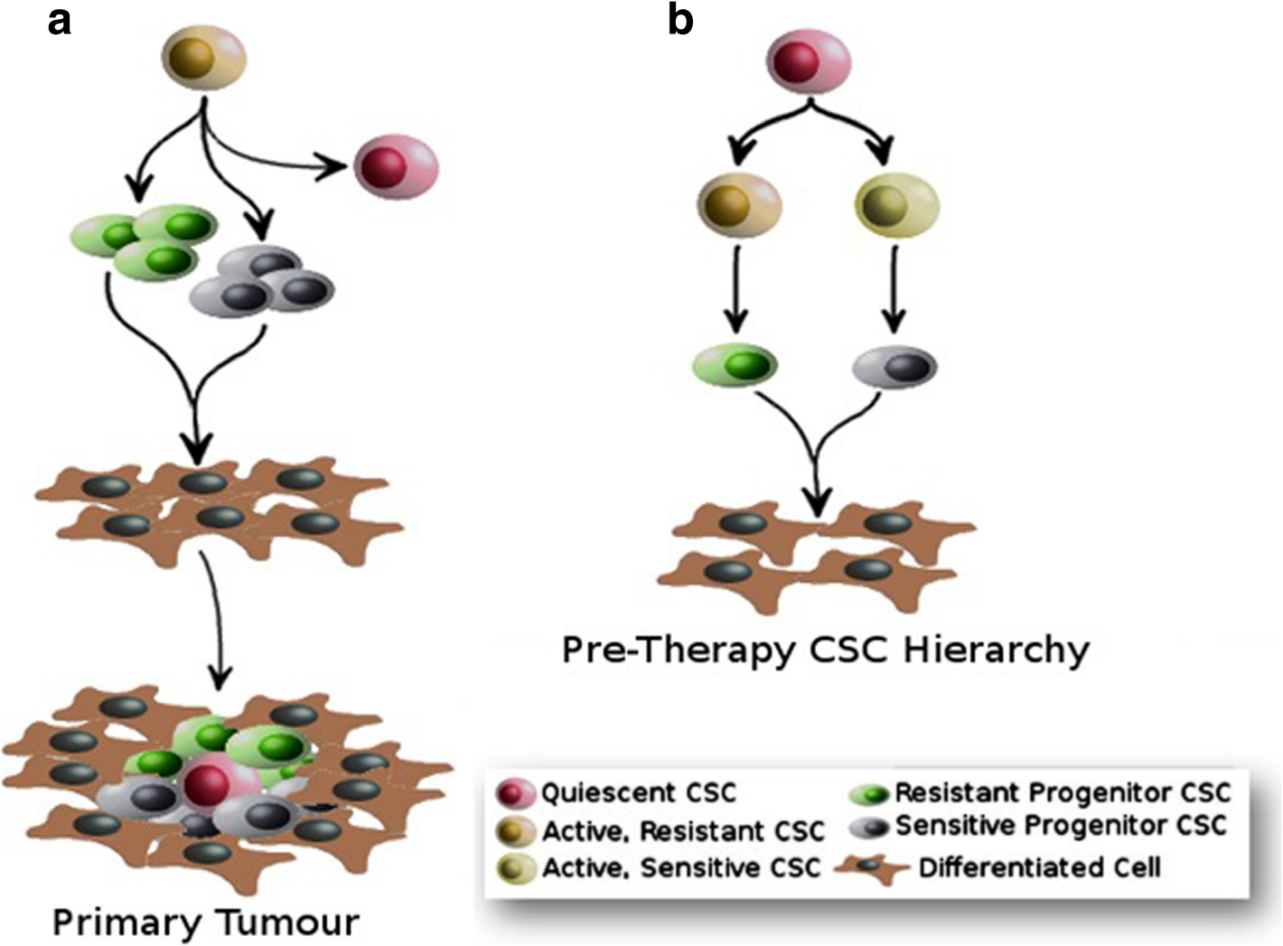
The Role of the CSC Hierarchy in Tumourigenesis. **a** Cancer Stem Cell (CSC) Theory indicates that tumourigenesis begins with rapid proliferation of stem cell-like tumour-initiating cells known as CSCs (*Orange*). Once a pool of CSCs has been established, less-potent ‘Progenitor CSCs’ are produced via differentiation, which our data indicate can be sensitive (*white*) or (*green*) resistant to conventional cancer therapies. These Progenitor CSCs differentiate to produce the mature cells (Brown) that constitute the bulk of the primary tumour. As the tumour becomes established, active CSCs (*Orange*) can enter a dormant state known as Quiescence (*Red*). **b** We propose an alternative model for hierarchical CSC structure where both CSCs and Progenitors can be sensitive or resistant to standard cancer therapies. Clinical-targeting of all CSC and Progenitor types is likely to compromise tumourigesis, which is an attractive clinical strategy. However, to efficiently treat refractory disease, this model suggests that it may be important to identify, model and target the specific therapy-resistant component(s) of the CSC hierarchy

In 2001, a seminal article by the Weissman group highlighted the need to develop CSC-targeting strategies to complement existing anti-cancer treatments [25]. It was proposed that the persistence of CSCs post-intervention was a likely explanation for recurrence. In the early days of CSC Theory, tumours were modelled as broadly consisting of a small population of tumour-initiating CSCs, surrounded by their differentiation progeny, which formed the bulk of the tumour. However, more recently this has been complicated by an appreciation that CSCs, like SCs, are organised hierarchically [Reviewed in 26]. The organisation of CSCs as Stem-Progenitor-Differentiated cell hierarchies was first described in LSCs [17]. However, in the 20 years since, relatively few hierarchies were characterised in specific malignancies. This was primarily due to the difficulty associated with identification and hierarchy-elucidation of specific CSCs from the heterogeneous population of CSCs found in tumours. Thankfully, rapid advances in technologies such as flow cytometry in recent years have permitted elucidation of CSCs in cancers such as skin, glioblastoma and liver [28–31]. Current models indicate that CSCs are organised as tree-like hierarchies (Fig. 1). At the top of the CSC hierarchy, the ‘Apex CSC’ is believed to spend very little time in an active state and, instead, primarily resides in a quiescent G_0_ state outside the cell cycle. In contrast, quiescence does not appear to be a property of progenitors CSCs. Mechanistically, it is believed that tumour-initiation involves three different stages (Fig. 1). First, apex CSCs enter a highly proliferative state, which results in the production of a population of lower potency Progenitor CSCs that is sufficient to continue tumour development. Secondly, apex CSCs enter quiescence while CSC progenitors undergo extensive asymmetric division to produce the mature, specialised cells that constitute the bulk of the tumour. Finally, CSC progenitor function slows while the tumour enters a process of maturation, where differentiated cell structures such as vasculature are completed. This may indicate a link between CSC activity and tumour grade: tumours that have not entered this maturation stage present as higher grade malignancies and are associated with worse prognosis due to their more proliferative phenotype.

### Ovarian cancer hierarchy

An interesting and illustrative example of the complex organisation of CSCs as hierarchies is the elucidation of these aspects of ovarian cancer in recent years. To date, several groups have described ovarian CSCs (ovCSCs [32–36]). However, many of these ovCSCs express different CSC markers, which reflect their identification in different models of a malignancy that is now considered to be a collection of diseases. For example, Chen et al. [35] and Silva et al. [33] have described CD44^+^/CD117^+^ and ALDH^+^/CD133^+^ ovCSCs, respectively (CD: ‘Cluster of Differentiation’; ALDH: Aldehyde Dehydrogenase). Subsequently, several studies have described ovCSCs based on comparison of cells expressing specific markers [32–36]. Despite the diverse nature of the markers, many ovCSC studies were combined in a consensus model for ovarian cancer proposed by Burgos-Ojeda et al. [37]. In recent years, the first ovCSC hierarchy has been described [33, 38]. In this work, single cell asymmetric division (SCAD) assays [Reviewed in 1] were used to describe a four-population hierarchy based on the expression of the stem cell markers ALDH and CD133. In this Stem-Progenitor-Differentiated cell model, highly-tumourigenic ALDH^+^/CD133^+^ CSCs can produce less-tumourigenic ALDH^+^/CD133^−^ Progenitor CSCs, which can in turn produce non-tumourigenic ALDH^−^/CD133^−^ Differentiated CSCs.

## Cancer stem cells and therapy-resistance

In early CSC Theory there was already an appreciation that CSCs were therapy-resistant [25]. The clear implication from this was that therapy-resistant CSCs could survive clinical intervention to regenerate recurrent disease through their tumour-initiation properties. It was proposed that therapy-resistant CSCs should be targeted in combination with conventional interventions as part of an overall anti-cancer strategy [25]. In subsequent years, this was largely interpreted as an indication that all CSCs were broadly resistant to all therapies. In the absence of models of CSC hierarchies, this was a reasonable view. This model was furthered by considerable evidence across many malignancies that indicated that whenever a CSC was identified, it displayed therapy-resistant properties. For example, it was found that cells positive for the CSC marker ALDH isolated from lung cancer cells lines demonstrated a high resistance to multiple chemotherapeutic agents (Cisplatin, Gemcitabine, Vinorelbine, Docetaxel, Doxorubicin and Daunorubicin) when compared to ALDH^−^ cells [39]. These chemoresistant CSCs can then re-establish the tumour following chemotherapy. However, it is now understood that tumours are composed of one or more CSC hierarchies, which are composed of multiple CSC-types [40, 41]. With the development of improved analysis tools, new questions can be asked. For example, it is important for clinical intervention that the relative therapy-resistance properties of different types of CSC within the tumour are assessed. An improved understanding of the relationship between CSC hierarchies and therapy-resistance can only aid the development of improved technologies. For example, it is established that SCs and CSCs from the same tissue type share many of the same self-renewal and differentiation regulatory mechanisms, which makes it difficult to target CSCs without damaging the non-malignant SC pool [42]. In a striking example of this, inhibition of Wnt Signalling as an anti-cancer treatment had devastating effects on Wnt-regulated normal development in pre-clinical studies [41]. A better understanding of the relationship between CSCs and therapy-resistance is needed to target CSCs specifically.

### Cancer stem cell hierarchies pose additional considerations for therapy-resistance

**Fig. 2:**
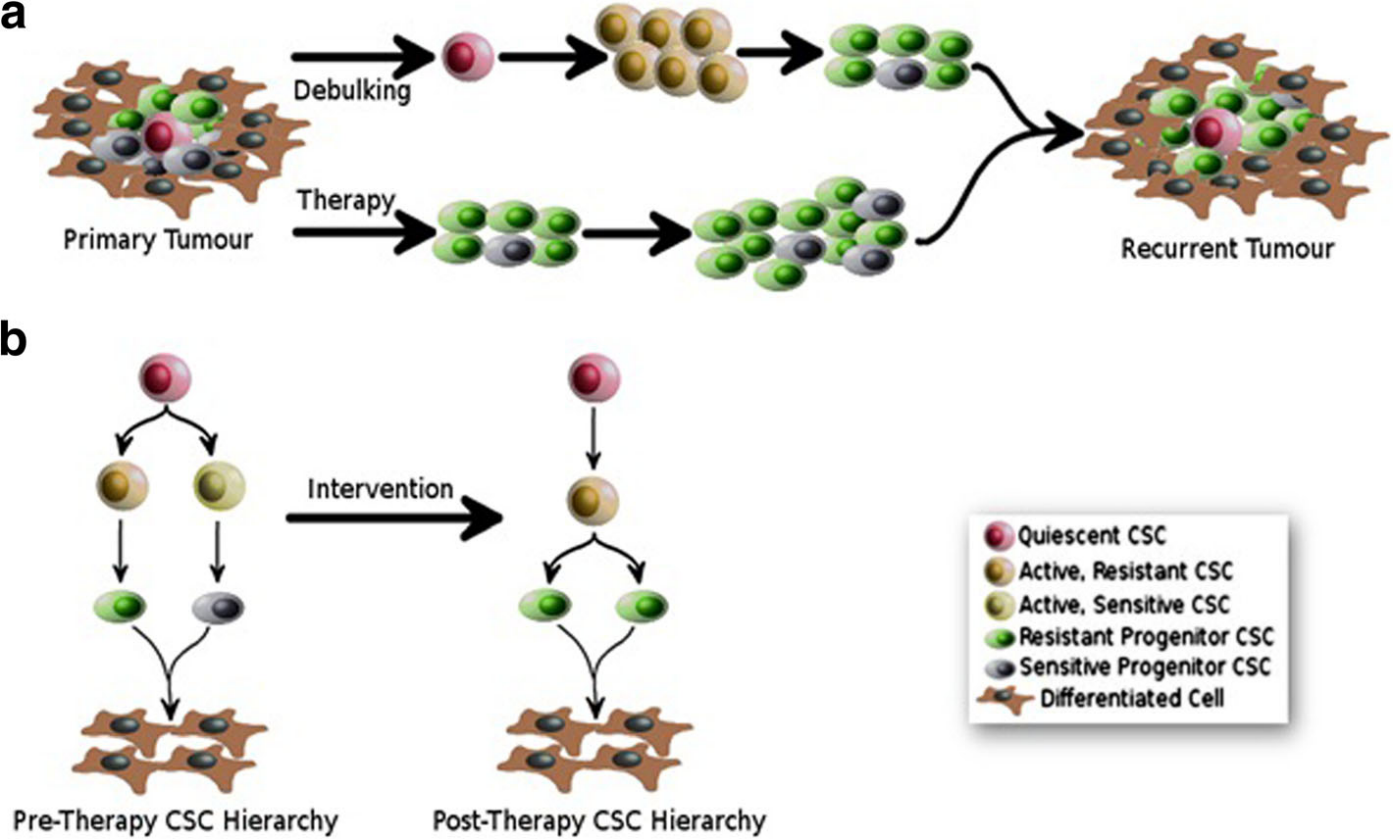
Clinical Implications of a Dynamic CSC Hierarchy Model. The model shown proposes that CSC hierarchies are altered by and adapt to clinical intervention, which poses additional consideration for clinical targeting of CSCs. **a** Many malignancies are treated by surgical removal of the tumour mass (debulking) and/or therapy (chemotherapy, radiation-therapy etc.). Contemporary CSC Theory suggests that debulking may cause an awakening of quiescent CSCs (*Red*). In parallel, anti-cancer therapies are likely to kill off therapy-sensitive CSCs (*Yellow, White*). This model proposes that this is likely to result in the formation of a recurrent tumour that is dominated by therapy-resistant CSCs (*Orange*) and Progenitors (*Green*). **b** This model proposes that CSC hierarchies are dynamic, particularly when challenged with therapeutic interventions. As a result, identification and targeting of specific therapy-resistant components within the malignancy may improve the treatment of recurrent disease

Modelling hierarchical CSC organisation is challenging. As a result, the relationship between CSC hierarchies and chemoresistance has, arguably, not been studied, which leaves several key questions open [1]. Specifically, these key questions are based upon our hypothesis that specific properties such as chemoresistance may be due to one or more specific members of a CSC hierarchy (Fig. 2). For example, which cell do we need to target: therapy-resistance might be a property of the apex CSC or the progenitor cell, or both? Are CSCs inherently resistant or do they adapt to therapy over time? How can we identify and specifically target therapy-resistant CSCs?

When considered in the context of the hierarchical organization of CSCs, the CSC(s) responsible for therapy-resistance must be characterized as either apex CSCs or progenitor CSCs, as this will determine the logical targeting strategy. Where these are apex CSCs, a direct target strategy is appropriate, as this should compromise the entire hierarchy. However, where these are found to be progenitor CSCs, the apex CSC that produces ‘therapy-resistant CSCs’ must also be identified, studied and targeted. By targeting both cell types simultaneously, treatments would ensure the removal of the CSC/progenitor responsible for therapy-resistant disease and the apex CSC responsible for their replacement. It is also important to note that the removal of active CSCs via chemotherapy stimulates quiescent CSCs into activity to drive further tumourigenesis [44, 45], Fig. 2]. To address this challenge, ideally both the active and quiescent CSC populations should be targeted. However, quiescent cells are by definition difficult to identify, isolate and study. An alternative for future treatments is a ‘Proliferate to Kill’ strategy, which aims to stimulate the quiescent CSC population, forcing them to re-enter the cell cycle, thus exposing them to standard therapeutic intervention [1].

## CSCs employ a collection of therapy-resistance mechanisms

The description of CSC therapy-resistance mechanisms in different malignancies in recent years [46] suggest a generalised vision of CSC therapy-resistance composed of five components: A) Quiescence; B) Detoxification/Multi-Drug Resistance (MDR); C) Repair of damaged DNA; D) Survival; E) Adaptation. As it emerges that these traits can be shared by CSCs from different malignancies, it is important to consider the nature of the underlying biology driving these mechanisms. One prospect is regulation by stemness signalling pathways such as Wnt and Notch, which are known to regulate several key mechanisms in many different types of SC and CSC. We propose that it would be prudent to consider each generalised set of mechanisms, which are now discussed in detail, when generating CSC models and designing clinical targeting strategies.

### Quiescent CSCs are insusceptible to conventional therapies

Contemporary SC Theory indicates that SCs are organised as hierarchies and that the apex SC at the top of the hierarchy can become quiescent [44, 47]. As quiescent cells reside outside the cell cycle they are broadly insusceptible to conventional therapies, which target the mitotic mechanisms of rapidly-dividing cells [48]. As such, ‘Quiescence’ is the first CSC therapy-resistance property. In more recent years, the description of two further mechanisms, DNA-monitoring and DNA repair during quiescence, offers further possibilities for the complexity of CSC therapy-resistance. In the first set of studies it has been shown that, rather than simply being inactive, quiescent SCs actively monitor DNA damage [49]. The strongest evidence for this mechanism is the demonstration that loss of key cell cycle regulators such as p53, p21, p57 or the Retinoblastoma protein impairs quiescence [50–53]. In addition, two related but separate studies have indicated that quiescent SCs are primed for re-entry in to the cycle in a process that involves DNA repair. Specifically, SCs have been shown to transition from G_0_ to G_1_ of the cell cycle via a ‘G_ALERT_’ state that is primed for rapid cell cycle re-entry in response to stress [54]. Complementing this study, it has recently been shown that quiescent HSCs can accumulate DNA damage and that many of these damaged HSCs are repaired during cell cycle re-entry rather than being signalled to undergo apoptosis [55]. These studies suggest that the quiescent CSC may possess a collection of DNA repair mechanisms that offer protection against therapeutic intervention. Despite the difficulty associated with modelling non-dividing cells, there are indications that quiescent stem-like populations contribute to at least some tumours. For example, in primary ovarian tumours it was demonstrated that CD24^+^ CSCs were slower proliferating but more tumourigenic than bulk cells, suggesting a quiescent, tumourigenic CSC population [56]. In addition, quiescent CSCs that survive chemotherapy can re-enter the cell cycle and re-establish the tumour [44, 57, 58].

### CSCs can detoxify in response to chemotherapy via multi-drug resistance efflux pumps

It is now established that many CSCs possess MDR mechanisms that function to detoxify the cell in response to chemotherapy. Multi-Drug Resistance is a collective term for efflux mechanisms that allow chemotherapy drugs to be pumped out of the cell before DNA damage can occur [59]. The best characterised MDR proteins are the members of the ‘ABC (ATP-Binding Cassette) transporter’ family, which were originally described in bacterial anti-biotic resistance [60]. It was subsequently found that many cell types possess ABC transporters. ABC transporters have been described in many types of cancer cells, where they act to efflux a wide array of chemotherapeutic drugs [61–63]. Furthermore, ABC transporters have been shown to be associated with chemoresistance of CSCs in several malignancies, including ovarian, breast, colon, and non-small cell lung cancers [64–67]. For example, the ABC transporters ABCB1 and ABCG2 have been associated with drug resistance in ovCSCs [64, 68]. As such, MDR is the second CSC therapy-resistance mechanism. Unfortunately, attempts to develop clinical-targeting of MDR mechanisms have not been successful to date [69, 70]. It appears that targeting specific ABC transporters leads to activation of redundant ABC transports to continue the MDR mechanism, which has hindered their exploitation as clinical targets [71].

### CSCs repair therapy-induced DNA damage via an enhanced DNA damage response

The third broad category of CSC therapy-resistance mechanism is the repair of DNA damage induced by chemotherapy and/or radiotherapy. In addition to those DNA repair mechanisms expressed by quiescent CSCs (described above), active cells can elicit a ‘DNA Damage Response’ (DDR [Reviewed in 72, 73]). There is now a broad appreciation that many therapy-resistant CSCs display elevated capacity for detection and repair of DNA damage, which allows them to survive via resistance to DNA damage induced by cancer therapies. Platinum-based chemotherapy is based upon forcing the formation of intra- and inter-strand DNA crosslinks (ICLs) upon the rapidly-diving cancer cell. ICL-formation disrupts chromatin structure, resulting in stalling of the replication fork and activation of several DDR pathways. In sensitive cells, the formation of multiple ICLs is beyond the cells DDR capacity. As a result, unrepaired DNA lesions cause cell-cycle arrest via apoptosis either directly or following DNA replication during the S phase of the cell cycle [74]. Pathways that have been shown to be involved in platinum-induced DNA damage include the Nuclear Excision Repair (NER) and the FA/BRCA pathway, each of which are now described in detail below. Similar DDR mechanisms are responsible for resistance to DNA-damage induced by radiotherapy. In contrast, taxane-based chemotherapies target microtubule dynamics during mitosis. Targeted microtubules cannot be repaired by the cell, which results in apoptosis via the G_2_/M checkpoint. Taxane-resistance is due to mutations in the tubulin sub-units that comprise microtubules [71], rather than therapy-resistance mechanisms. As such, taxane-resistance is not discussed in this review.

#### Nucleotide excision repair

Intra-strand crosslinks, the most abundant lesion, are repaired via NER [75], a DDR mechanism that is often overexpressed in therapy-resistance disease. Mechanistically, NER involves recognition and excision of single-strand DNA damage. The remaining undamaged single-strand DNA is used as a template for DNA synthesis, which is followed by ligation to complete the DDR process. Excision is facilitated by the ERCC1 (excision repair cross – complementation group 1) protein. Overexpression of ERCC1 contributes to platinum-resistance in many cancers, such as ovarian, non-small cell lung and testicular cancers [76–78]. Recent studies indicate a link between overexpression of NER proteins and to CSC chemoresistance [79]. However, this has not definitively been demonstrated. Overexpression of ERRC1 may be a mechanism of CSC therapy-resistance, as elevated mRNA and protein levels of ERRC1 have been shown in platinum-resistant oral CSCs [80]. However, Wang et al. [81] have demonstrated that NER protein levels are unrelated to platinum-sensitivity in ovarian cancer. Furthermore, knockdown of NER factors compromised NER efficiency, but caused only a minimal effect on platinum-sensitivity [81].

#### The fanconi anemia/BRCA DNA damage repair pathway

The detection and repair of ICLs also involves the cooperation of the Fanconia Anemia (FA) and Breast Cancer 2 (BRCA) pathways, which are now often referred to as the FA/BRCA pathway. In this mechanism, DNA damage is detected and removed by the FA pathway, and DNA synthesis subsequently completed by the BRCA-regulated homologous recombination (HR) pathway [72, 73]. FA is an uncommon disorder characterised by congenital abnormalities, progressive bone marrow failure, and cancer susceptibility [82, 83]. FA was found to be due to abnormalities in a novel DDR mechanism that became known as the FA pathway. Subsequently, the FA pathway was found to be associated with the development of cancers in FA patients [84] and therapy-resistance in cancer generally [85, 86]. The FA/BRCA pathway begins with recognition of ICLs by the FA complementation group complex (FANC), which is composed of multiple FA proteins (A, B, C, D1, D2, E, F, G, I, J, L, M and N). The connection between the FA pathways was originally highlighted by studies showing that FANCD1 and BRCA2, and FANCS and BRCA1 are the same genes. Upon ICL detection, the FA pathway is activated by the monoubiquitination of FANCD2 and FANCI. FANCD2 is subsequently targeted to the damaged chromatin site where it interacts with the BRCA2 protein, a key regulator of HR [Reviewed in 72, 73]. This interaction appears to be required for normal HR and ICL repair. The molecular relationship between monoubiquitinated FANCD2 and BRCA2 in the HR component of DDR is not known: it is speculated that the proteins may cooperate in the timed release of RAD51 at sites of DNA repair [87, 88].

Activation of the FA/BRCA pathway culminates in repair of ICLs, UV-induced dimers, and double-strand breaks [89, 90], which plays an important role in the acquisition of drug resistance in many cancers, such as multiple myeloma, glioma, cervical and ovarian cancer [91–94]. Recent studies indicate that overexpression of FA/BRCA pathway proteins is also linked to CSC chemoresistance. For example, Meng et al. [34] demonstrated that platinum resistant ALDH^+^ ovarian cancer cells express elevated levels of FA/BRCA DNA repair proteins. It is well-established that loss of function BRCA mutations leads to increased susceptibility to the development of cancer, particularly in hereditary disease. In these cases, cancer cell therapy-resistance becomes dependent upon related, functional DDR mechanisms, which can be targeted to improve clinical outcomes [Reviewed in 95]. For example, inhibition of DDR components known as ‘PARPs’ (poly ADP ribose polymerases) can enhance therapy-sensitivity in some patients [Reviewed in 95]. More recently it has been shown that functional over-expression of BRCA is associated with therapy-resistance in non-inherited disease [96–99]. Some evidence is emerging that FA/BRCA inhibitors such as the FDA approved Bortezomib can improve chemoresponse in otherwise refractory disease [100, 101].

### CSCs employ anti-apoptotic mechanisms to promote survival in response to clinical intervention

The accumulation of substantial DNA damage results in the activation of apoptotic mechanisms, which must be inhibited in therapy-resistant cells. For example, it is now well-established that evasion of apoptosis is one of the major mechanisms of ovarian cancer associated platinum-resistance [102, 103]. Anti-apoptosis mechanisms contribute to therapy-resistance directly by blocking cell death and indirectly by providing time for enhanced DDR mechanisms to repair therapy-induced DNA damage. These anti-apoptotic mechanisms include the extrinsic and intrinsic pathways, and the tumour suppressor protein p53, each of which will now be discussed. Collectively, these represent a fourth, pro-survival mechanism for CSC therapy-resistance.

#### The extrinsic apoptotic pathway

The extrinsic apoptotic pathway is characterised by detection of death signals by ‘Death Receptors’. Death receptors are cell-surface expressed, and belong to the superfamily of tumour necrosis factor receptors (TNF-R) that are activated by TNF family ligands. The best-characterized death receptors include FAS (APO-1/CD95), TNF receptor 1 (TNFRI), TNF-related apoptosis-inducing ligand-receptor 1 (TRAIL-R1) and TRAIL-R2. Upon stimulation by the FAS ligand, FAS trimerises, which allows intracellular binding of the adaptor-protein FADD to the receptor. FADD in turn serves to recruit pro-caspases-8 and −10 into the complex, which ultimately activates downstream caspases, leading to apoptosis [104, 105]. Cancer cells have evolved numerous strategies to resist drug-induced cell death via the extrinsic pathway. For example, surface expression of death receptors have been shown to be downregulated or absent in drug resistant tumours, including platinum-resistant ovarian cancer [106]. Upregulation of FAS has been shown to reverse platinum-resistance in ovarian cancer [107]. While FAS plays a pro-apoptotic role in SCs, it appears to promote survival in CSCs [108]. Furthermore, upregulation of the adaptor-protein FADD has been shown to sensitise ovarian cancer cells to platinum treatment [109]. Although little is known yet about evasion of apoptosis as a possible CSC chemoresistance mechanism, data indicates that CSCs resist cell death via the extrinsic pathway. For example, an upregulation of TRAIL-R1 leads to chemoresistance in colon CSCs [110]. Several mechanisms have been identified in mammalian cells for the induction of apoptosis. These mechanisms include factors that lead to perturbation of the mitochondria leading to leakage of cytochrome C or factors that directly activate members of the death receptor family. Epothilones are a new group of compounds with action mechanisms similar to taxanes. In an ovarian cancer model Epothilone A and B have been shown to induce cell death via a decrease in mitochondrial membrane potential [111]. This may represent a future clinical strategy.

#### The intrinsic apoptotic pathway

The intrinsic apoptotic pathway induces cell death by affecting mitochondrial permeability, which leads to the release of cytochrome C to activate caspases. Among its many regulators, the pathway is primarily regulated by members of the BCL-2 family [112, 113]. The protein BCL-2, the founding member of the BCL-2 family, primarily mediates its pro-survival effects by binding to the pro-apoptotic partners BCL2-associated-X-protein (BAX) and BCL-2 homologous antagonist killer (BAK), which decreases release of cytochrome C from the mitochondria. BCL-2 family members are overexpressed in many solid tumours and have been linked to tumourigenesis, cancer cell survival and chemoresistance [109, 114, 115]. Recent studies show that BCL-2 overexpression is also linked to CSC chemoresistance. For example Madjd et al. [116] showed that the protein BCL-2 is highly expressed in CD44^+^/CD24^-/low^ breast CSCs. To date, little is known about the mechanism, but CSC research suggests that these proteins can affect chemoresistance through induction by other signalling pathways required for CSC survival. For example, Ma et al. [117] demonstrated that BCL-2 induction by AKT1 may be a mechanism by which CSCs can mediate chemoresistance.

#### The role of p53 in apoptosis

The tumour suppressor p53 acts as the overall regulator of the activation of apoptotic processes in response to the detection of unrepairable DNA damage [118, 119]. Like BRCA, ‘loss of function’ p53 mutations are associated with the development of various malignancies, while its over-expression in cancers with functional DDR mechanisms is associated with therapy-resistance. In addition, numerous chemotherapy drugs exert their function through targeting p53-related signalling pathways. Therapy-induced DNA damage leads to activation of p53, which binds to the regulatory sequences of a number of target genes to initiate a program of cell cycle arrest and DDR. If the drug-induced damage cannot be repaired completely, over-activation of p53 leads to tumour growth stagnation or even apoptosis via the induction of the intrinsic and extrinsic apoptosis pathways [120–122]. Loss of p53 function occurs during the development of most, but not all, tumour types. This results in tumour cells being able to escape drug-induced apoptosis [122]. A downregulation of p53 has been shown in platinum-resistant ovarian cancer [109]. However, recent studies show an alternative mechanism where p53 overexpression is linked to CSC chemoresistance. For example, it was shown that overexpression of p53 sensitises glioblastoma CSCs to drug treatment trough enhanced apoptosis [123].

### CSC hierarchies can adapt to survive clinical intervention

The principles governing the relationship between CSC hierarchies and therapy-resistance are poorly understood. Our most recent work has highlighted that therapy-resistance is not the property of all CSCs within a hierarchy: therapy-resistance can be CSC- and drug-type specific (Ffrench et al. Unpublished). Furthermore, we have found that progenitor CSCs can adapt to platinum-treatment by altering their potency (producing a different cell type via differentiation). This data indicates that, in at least some cases, CSC models contain more than one type of CSC, some of which are therapy-sensitive and some therapy-resistant. This data suggests a model (Fig. 2) where the treatment-naïve CSC hierarchy can contain therapy-sensitive and therapy-resistant CSCs. Post-intervention, sensitive CSCs are killed off, which results in a recurrent tumour and CSC hierarchy with increased numbers of therapy-resistant CSCs. In addition, removal of the tumour by surgical de-bulking may awaken quiescent CSCs to synergistically enhance the production of the therapy-resistant recurrent malignancy. This may explain why initial pre-clinical success has not broadly translated to the clinic (Fig. 2). As such, it is important to identify, study and target the specific CSCs responsible for specific types of therapy-resistance within specific malignancies.

### Altered CSC-related signalling pathways in therapy-resistance

SC function is regulated by a number of signalling pathways such as Wnt and Notch [1], which are commonly dysregulated in therapy-resistant CSCs [124]. However, targeting of these pathways can have adverse effects on similarly-regulated non-malignant SC pools. The involvement of stemness signalling pathways such as Wnt and Notch in CSCs from multiple different types of malignancy is now described and suggests an important role in CSC therapy-resistance. It is tempting to speculate that these stemness signalling pathways may facilitate coordinated CSC therapy-resistance mechanisms such as quiescence, detoxification (MDR), repair (DDR), survival (anti-apoptosis) and adaptation.

The Wnt signalling pathway has been shown to play an important role in the maintenance of SCs and lineage differentiation in a wide array of tissues and organs [125]. Altered expression of this pathway has been shown in many cancers, such as ovarian, breast and colon [126–128]. While the precise mechanism has not been fully elucidated, elevated levels of Wnt Signalling modulator β-catenin have been shown to actively contribute to platinum-resistance in ovarian cancer cells [129]. Hepatic CSCs have been shown to exhibit enhanced platinum-resistance that could be reversed by lentiviral microRNA knockdown of β-catenin [130]. It is not clear how Wnt signalling mediates CSC chemoresistance but upregulation of ABC transporters presents one potential mechanism: ABCG2 resistance to both platinum and taxanes could be reversed by β-catenin siRNA knockdown in c-kit^+^ ovCSCs [131]. Furthermore, it was shown that inhibition of the Wnt signalling pathway, in combination with platinum, induced cytotoxicity and cell cycle arrest in a higher percentage of primary ovarian samples than with single drug treatment [126]. Because of the molecular similarity between SCs and CSCs from the same region of the body it is very difficult to target CSCs without damaging the non-malignant SC pool, a side-effect that would have devastating growth and repair consequences for the patient. In a stark example of this, Wnt inhibition as an anti-cancer treatment has been shown to have devastating effects on Wnt-regulated normal intestine development in pre-clinical studies [43].

The Notch signalling pathway plays a role in both CSC maintenance and drug resistance in many malignancies [Reviewed in 1]. For example, Notch proteins have been identified to be upregulated in ovCSCs [132]. Furthermore, in the same study a γ-secretase inhibitor (GSI), which inhibits Notch signalling, was shown to erase CSCs and increase tumour sensitivity to platinum-agents. Most importantly, it was found that platinum/GSI combination compared to single treatment was more effective to eliminate both CSCs and the bulk of tumour cells, indicating that a dual combination targeting both populations is needed for tumour eradication. In addition, the group found that the Cisplatin/GSI combination therapy has a synergistic cytotoxic effect in Notch-dependent tumour cells by enhancing the DNA-damage response, G_2_/M cell-cycle arrest, and apoptosis [132]. The superior effectiveness of a combination of chemotherapy drug and Notch-targeting CSC-inhibitor (GSI) compared to monotherapy in an otherwise refractory disease, has also been reported in pancreatic metastasis [133]. As such, it is emerging as an early paradigm for how CSC-targeting should be conducted as part of an overall treatment [1].

In the absence of other evidence, stemness signalling pathways appear to be likely candidates for a role in coordinating the multiple responses of therapy-resistant CSCs to clinical intervention. We propose that all of the mechanisms described in this section should be considered during analysis of CSC models. An improved understanding of the mechanisms of CSC therapy-resistant may facilitate development of novel treatment strategies. This is discussed in detail later. However, before they can be analysed, CSC models must be built.

## CSC discovery: identifying the therapy-resistant components of CSC hierarchies

While it is well-accepted that CSC-targeting must be assessed as a novel therapeutic avenue, little progress has been made in improving survival rates in the clinic. We believe that this is partially due to our failure to identify specific therapy-resistant component in the context of CSC hierarchies. We hypothesise that it cannot simply be assumed that each component of a CSC hierarchy contributes to therapy-resistance equally. Addressing this, we have recently built a novel, four-component CSC hierarchy and assessed platinum- and taxane-resistance in each, individual CSC type. Our data indicate that platinum-resistance is the property of only one CSC-type within that hierarchy and that all four components are taxane-sensitive. Additionally, we found that repeated exposure to platinum results in an altered hierarchy with altered plasticity, with a different CSC type taking the ‘Apex’ position at the top of the hierarchy (Ffrench et al. Unpublished). These findings impose new considerations upon CSC research, which are now discussed in the context of our perspective on CSC Discovery and clinical targeting.

### CSC discovery

We propose that therapy-resistant CSC Discovery should be conducted in paired therapy-sensitive and therapy-resistant models. In our opinion, this is one of most over-looked factors in CSC Discovery and is a large determinant of success [1]. For a detailed description of our approach to CSC Discovery the reader is referred to [1]. Briefly, CSC Discovery should begin with a model that addresses a clinical problem. For example, if a particular malignancy is characterised by high levels of mortality due to platinum-resistance, CSC Discovery is best undertaken in paired cell models of platinum-sensitivity and platinum-resistance in that malignancy. Many available cell line models are therapy-naïve and can be easily rendered therapy-resistant by exposure to increasing doses of the therapy over time. While these cell lines are not a perfect facsimile of in vivo disease, many studies have demonstrated that they are readily available sources for CSC Discovery. Broadly, CSCs have been successfully identified through screening for SC markers such as ALDH and ‘Cluster of Differentiation’ (CD44, CD133 etc.), and SC properties such as efflux (Hoechst dye efflux ‘side population’ assay) and non-adherent growth (‘spheroid formation’) assays. Once identified and validated, these CSCs are available for in vivo validation in clinical samples. Subsequently, CSC hierarchy relationships can be elucidated as now described.

### Building a therapy-resistant hierarchy

The rapid advances in flow cytometry-based technology in recent years mean that building CSC hierarchies is now much more achievable than it has been in the past. In this regard, we have found the SCAD assay particularly useful [1]. In this assay, single cells positive and negative for identified SC markers are allowed to form colonies, which are then tested for the presence of the marker: where both positive and negative cells are found in the colonies, this is suggestive of SC properties. Importantly, SCAD assays can easily be scaled to include two or more markers. For example, where two SC markers (A and B) are identified in screens, a four component hierarchy (A^+^/B^+^, A^−^/B^−^, A^+^/B^−^, A^−^/B^+^) can easily be built. Characterising of the colonies formed allows the relationship between each CSC type to be established, and an accurate model of the CSC hierarchy to be identified, which may include the apex CSCs, and inter-related and independent progenitors [1]. For example, and SCAD approach has recently been used to describe an ovCSC hierarchy based on the expression of ALDH and CD133 [38]. In this model, highly tumourigenic ALDH^+^CD133^+^ CSCs give rise to somewhat less tumourigenic ALDH^+^CD133^−^ CSC/progenitors, which in turn give rise to non-tumourigenic ALDH^−^CD133^−^ cells. Once validated in standard xenograft-formation assays, the platinum-resistant component of this hierarchy could be identified, and the mechanism identified as now described.

### Identification of the therapy-resistant component of a CSC hierarchy, and its mechanism

The standard approach to the identification of therapy-resistant cells is to test populations for survival following treatment with chemotherapy or radiotherapy. As such, measuring the relative contribution to resistance of each cell type in a CSC hierarchy appears to be a daunting task. However, flow cytometry’s ability to accurately measure the relative proportion of sub-populations within a CSC hierarchy provides an elegant solution. This approach can be adapted for CSC hierarchy research purposes by simply assaying the post-treatment CSC hierarchy for the presence of its original SC markers via flow cytometry. This approach easily facilitates quantification of the relative therapy-resistance of each individual CSC type within the hierarchy, which is a strong indication of their proportional contributions to therapy-resistance. A therapy-sensitive model can be assessed pre- and post- acute treatment, and the resistance of specific CSC populations highlighted by an increase in their respective proportions. Furthermore, chronic treatment of the sensitive model over time can be used to generate a paired resistant model, which can be reanalysed before and after acute treatment. Such a matrix of comparisons will point towards the identity of the intrinsically and adaptively resistance cell populations within the models being investigated.

This analysis can be carried out on both the entire hierarchy or individual CSC types present in sufficient numbers to allow purification for assays. As a caveat, we have noted that the quantitative tolerance of resistant CSC types can be higher when assayed in isolation compare to within the hierarchy as a whole. This is likely due to the absence of pro-apoptotic signalling from therapy-sensitive CSCs that are absent when a therapy-resistant CSC is assayed in isolation. Once identified, the specific mechanisms expressed by individual therapy-resistant CSC types can be identified using standard gene expression assay or RNAseq approaches. For example, our most recent work has highlighted elevated expression of MDR, DDR and anti-apoptotic mechanisms in platinum-resistant CSCs compared to platinum-sensitive CSCs from the same hierarchy (Ffrench et al. Unpublished). As a final consideration, we have found that specific MDR, DDR and anti-apoptotic mechanisms are expressed in treatment-naïve and platinum-adapted models, as discussed above. It is clear that this observation would likely have been missed had our work been undertaken in a single model. This is strong evidence for the undertaking of CSC Discovery in paired therapy-sensitive and therapy-resistant models.

## Clinical perspective: towards targeting of therapy-resistant CSCs

Early CSC Theory highlighted several principles on which clinical targeting of CSCs should be based. At the time, there was great hope that CSC targeting in the clinic could dramatically improve the prognosis of refractory disease. In spite of this, the translation of CSC-targeting to the clinic has not been as efficient as had been hoped. In this section we will describe some of the clinical implications of our contemporary understanding of CSC Theory. In particular, we will discuss how our clinical-targeting strategies must allow for the model of CSC hierarchies as moving, rather than static, targets.

### Clinical considerations from early CSC theory

CSC theory as presented by Reya et al. [25] highlighted several clinical implications of relevance to our understanding of how CSCs influence therapy-resistance. Firstly, CSCs were identified as clear clinical targets due to their role in primary, metastatic and recurrent disease. Secondly, low numbers of CSCs persisting post-intervention were sufficient to drive recurrent disease. As the CSCs responsible for this repopulation were by definition therapy-resistant, it was suggested that this property would be passed on to the entire progeny of the tumour reinitiating CSCs. This theory, therefore, could account for the higher levels of therapy-resistance found in recurrent disease compared to primary tumours. Thirdly, CSCs were originally thought to be rapidly-dividing and thus particularly susceptible to standard interventions, which are designed to target rapidly dividing (cancer) cells. The fact that this was not the case in the clinic provided evidence that CSCs possessed strong therapy-resistance properties, which were postulated as mechanisms such as enhanced MDR and DDR. Finally, one of the most contentious suggestions at this point was that CSCs were not simply one of many different types of tumour-initiating cell but were the only known tumour-initiating cell. Indeed, to this day an efficient, tumour-initiating cell de-void of stem cell properties has not been reported to our knowledge. This implication presents CSCs as the primary culprit for circulating tumour cells that facilitate metastasis. As such, targeting CSCs should produce the additional benefit of reducing metastasis. These concepts were widely accepted by the cancer research community, which held out great hope that CSC targeting could be developed as part of a combined cancer treatment strategy, which would be particularly suited to refractory disease. Strategically, it was believed that CSCs should be targeted during primary treatment to prevent metastasis and recurrence and the development of therapy-resistance.

This model suggested a strategy where cancer treatment could be improved by removing stemness properties from CSCs globally. This so called ‘Forced Differentiation’ approach has been best examined using differentiation morphogen all-*trans* retinoic acid (ATRA), which has being explored in a variety of haematological malignancies, including myelodysplastic syndromes, multiple myeloma and chronic myelogenous leukemia [134, 135]. Alone or in combination with other therapies, ATRA is also being investigated in a variety of solid tumours including prostate cancer, breast cancer and glioma [136–138]. Although pharmacological doses of retinoids have proven effective in the treatment of haematological malignancies [139], clinical trials in the prevention and treatment setting in a number of solid tumours have failed to show significant benefit to date [68, 140]. For example, clinical trials in breast cancer have shown moderate potential, but consistently low response rates of below 30% [141, 142]. This is one example that illustrates how inefficient CSC-targeting approaches have been to date. This suggests that targeting CSCs globally may not be an efficient strategy in all but a few, specific malignancies. In contrast, our contemporary understanding of CSCs suggest that specific targeting strategies may be more successful, which is now discussed in detail.

### Contemporary CSC theory suggests New considerations for clinical intervention

The important CSC-targeting principles highlighted by Reya et al. [25] provide the foundation upon which contemporary CSC Theory has been built. Early CSC theory suggested that tumour heterogeneity could be broadly divided in to two cell types, namely CSCs and differentiated cells. In the intervening years it has become clear that heterogeneity in at least some, and perhaps many or all, malignancies is due to the presence of multiple CSC types arranged as hierarchies [26]. There is clear evidence that the identification and targeting of a single CSC-type has potential as a clinical strategy. Perhaps the most striking example of this was described in ovarian cancer, where targeting of Notch signalling in ovCSCs resulted in platinum-based elimination of disease in an animal model of otherwise refractory disease [132]. However, a large collection of similar studies has not been produced in other malignancies. In addition, there have been few successes in the clinic. We propose that one of the key factors behind this failure is the complex hierarchical organisation of CSCs, which complicates clinical targeting. Rather than looking for a ‘needle in a haystack’, which was challenging enough, it appears that we must now locate and target a specific, clinically-relevant needle in a collection of similar needles within the haystack. Complicating this further, it appears that CSC hierarchies are altered by and adapt in response to our interventions, making them complex moving targets.

### Clinical implications of CSC hierarchy dynamics

Contemporary CSC Theory proposes that tumour heterogeneity is due to the presence of multiple CSC Hierarchies within a single tumour [26]. In addition to the concepts described here, evidence is emerging for other forms of plasticity such as the conversion of non-CSCs to CSCs phenotypes in defined circumstances [143]. This more complex view of in vivo CSC organisation has important considerations for clinical targeting. The most obvious principle is that the apex CSC(s) should be targeted in order to compromise the entire hierarchy. As discussed above, apex CSCs might only be readily targetable when they are active during early tumourigenesis, as targeting the quiescent state is currently unachievable. This suggests a strategy where targeting of CSC progenitors should substantially compromise tumourigenicity. However, as quiescent (apex) CSCs are largely insusceptible to anti-mitotic cancer treatments, targeting CSC progenitors is likely to result in an awakening of the quiescent CSC pool and rapid regeneration of the tumour (Fig. 2). This may partially explain why successful primary intervention can be followed months later by substantial, aggressive recurrent disease.

This model of recurrent disease is based upon an assumption that awakened quiescent CSCs reconstitute the primary malignancy as recurrent disease. However, in our lab we have observed that therapeutic intervention can alter the CSC hierarchy, which has important considerations for clinical targeting. Our data indicate that only some components of a CSC hierarchy are resistant to platinum. As a result, after only a few days the hierarchy is altered: the sensitive CSC populations have been dramatically reduced in size, leaving a proportionally larger resistant CSC population (Fig. 2). As a result, after only a few days of platinum treatment the CSC hierarchy observed post-intervention is substantially different to the treatment-naïve model (Fig. 2). As such, we hypothesise that CSC hierarchies within the tumour are not ‘fixed targets’: in terms of CSC organisation, the primary tumour can be quite different to the recurrent tumour. This may partially explain why primary disease can be sensitive and recurrent disease resistant to the same therapy.

## Conclusion

### Catching moving targets: elucidation and targeting of dynamic CSC hierarchies

Considerable advances need to be made before efficient, clinically-relevant targeting of entire CSC hierarchies can be achieved. To date, very little research has been carried out to elucidate the relationship between CSC hierarchies and therapy-resistance. We have argued that therapy-resistance within CSC hierarchies can be highly specific: therapy-resistance can be the property of a specific type of CSC and not a global property of the hierarchy; CSCs that are resistant to one type of therapy (e.g. platinum) can be sensitive to another (e.g. taxanes); CSC hierarchies can be altered as sensitive populations are killed off by our interventions; this can result in substantial alterations to the CSC hierarchy, including the moving of a different CSC type to the apex position. While these observations must be tested and validated in other malignancies, this suggests that a broader, more specific view of CSC Discovery and targeting may be more successful that global targeting approaches. This type of specificity is in line with the personalised medicine approach, which has been highly successful in the clinic in malignancies such as breast cancer.

Dynamic CSC hierarchies are best modelled using paired-models of sensitive and resistant disease that are specific for different therapeutic-interventions. The obvious application for this is in the treatment of therapy-resistant breast cancer. In recent years, breast cancer treatment has been dramatically improved by the identification of sub-types within the disease that respond best to specific therapies. However, in contrast, the understanding of the specific CSC hierarchies associated with each sub-type of breast cancer is sparse. The identification and targeting of the specific CSC hierarchies responsible for therapy-resistance in different types of breast cancer may facilitate the development of improved treatment strategies. In addressing this, it is important to note that the CSC hierarchies in each sub-type may be quite different from one another and altered post-intervention. In addition, the success in breast cancer suggests that the specific targeting of sub-types within malignancies can enhance outcomes in other refractory malignancies. It seems clear that there is great potential for the exploitation of the specificities of CSC types within sub-types of malignancy as both bio-markers for early detection and targets for the treatment of refractory disease.
